# Delafloxacin, Finafloxacin, and Zabofloxacin: Novel Fluoroquinolones in the Antibiotic Pipeline

**DOI:** 10.3390/antibiotics10121506

**Published:** 2021-12-08

**Authors:** Béla Kocsis, Dániel Gulyás, Dóra Szabó

**Affiliations:** Institute of Medical Microbiology, Semmelweis University, 1089 Budapest, Hungary; gulyas.daniel@med.semmelweis-univ.hu (D.G.); szabo.dora@med.semmelweis-univ.hu (D.S.)

**Keywords:** novel antibiotics, multidrug resistance, fluoroquinolones

## Abstract

Novel antimicrobial agents, approved for clinical use in past years, represent potential treatment options for various infections. In this review, we summarize the most important medical and microbiological features of three recently approved fluoroquinolones, namely delafloxacin, finafloxacin, and zabofloxacin. Delafloxacin possesses an anionic chemical structure, and represents broad-spectrum activity, as it targets both bacterial DNA gyrase and topoisomerase IV enzymes of gram-positive and gram-negative bacteria with equal affinity. Its molecular surface is larger than that of other fluoroquinolones, and it has enhanced antibacterial efficacy in acidic environments. Delafloxacin has been approved to treat acute bacterial skin and skin-structure infections, as well as community-acquired bacterial pneumonia. Finafloxacin has a zwitterionic chemical structure, and targets both DNA gyrase and topoisomerase IV enzymes. This enables a broad antibacterial spectrum; however, finafloxacin has so far only been approved in ear-drops to treat bacterial otitis externa. Zabofloxacin is also a broad-spectrum fluoroquinolone agent, and was first approved in South Korea to treat acute bacterial exacerbation of chronic obstructive pulmonary disease. The introduction of these novel fluoroquinolones into daily practice extends the possible indications of antibiotics into different bacterial infections, and provides treatment options in difficult-to-treat infections. However, some reports of delafloxacin resistance have already appeared, thus underlining the importance of the prudent use of antibiotics.

## 1. Introduction

Nowadays, the emergence and dissemination of multiresistant pathogens poses an ongoing challenge [1,2,3]. Increasing numbers of infections caused by antibiotic-resistant bacteria are being diagnosed worldwide, and the most well-known group of multiresistant pathogens is the ESKAPE group, namely *Enterococcus faecium*, *Staphylococcus aureus*, *Klebsiella pneumoniae*, *Acinetobacter baumannii*, *Pseudomonas aeruginosa*, and *Enterobacter* spp. [4]. These pathogens frequently develop resistance to various antibiotics, and are common causative agents of difficult-to-treat nosocomial infections, including bloodstream, wound, skin, and urinary tract infections (UTIs), as well as ventilator-associated pneumonia (VAP) [5]. Due to the limited number of effective antibiotics, infections caused by ESKAPE pathogens are associated with significantly high morbidity and mortality rates [6,7,8].

The need for novel potent antimicrobial agents is seen worldwide. The World Health Organization (WHO) released a priority list detailing the discovery and development of novel antibiotics [9]. On this WHO priority list, ESKAPE pathogens are listed among “critical” and “high” priority groups; therefore, development of antibiotics is urgently needed against these pathogens [9].

During the past years, huge efforts have been made towards the development of novel agents; several antimicrobial agents have been synthetized, and some of them are already approved for clinical use [10]. The Food and Drug Administration (FDA) approved plazomicin as a new aminoglycoside, which has been recommended for therapy of complicated urinary tract infections caused by multidrug-resistant Enterobacteriaceae [11]. Cefiderocol, a siderophore cephalosporin, has been approved for therapy of infections caused by carbapenem-resistant gram-negative bacteria [12]. Different combinations of beta-lactams and beta-lactamase-inhibitors have also been approved for clinical use, namely meropenem/vaborbactam, imipenem/cilastatin/relebactam, ceftazidime/avibactam, and ceftolozane/tazobactam [10,13,14]. New fluoroquinolones with enhanced antibacterial features are also under development, and some of them are marketed [15,16]. In this review we summarize the most important medical and microbiological features of three recently approved fluoroquinolones, namely delafloxacin, finafloxacin and zabofloxacin.

## 2. Fluoroquinolones

Fluoroquinolones are nucleic acid synthesis inhibitors, and their main targets are bacterial gyrase and topoisomerase IV enzymes. Fluoroquinolones were first synthetized in the 1970s and 1980s, and, later on, these agents were introduced into clinical practice [17]. The chemical structure of fluoroquinolones includes a common bicyclic quinolone ring; however, throughout the past decades, different substituents have been added to this common quinolone ring to achieve better tissue penetration and improved antibacterial efficacy [17].

Nalidixic acid and oxalinic acid are quinolone agents which lack the fluorine atom in their chemical structure. These agents were applied in clinical practice in the 1960s to treat urinary tract infections. However, these quinolone agents represented a narrow spectrum antibacterial activity, and nowadays these agents are not recommended for therapy [18].

Structural modifications through the addition of substituents on certain part of the quinolone ring yielded increased potency of agents, namely in terms of pharmacokinetic features and antibacterial spectrum. The most important substituents on the basic quinolone ring are a cyclopropyl or difluorophenyl in position C1, a fluorine in position C6, a halogen, methoxy, or fused third ring in position C8, and a piperazine in position C7. Fluoroquinolones targeting both gyrase and topoisomerase IV enzymes have broad-spectrum antibacterial effect [18]. 

These structural modifications of fluoroquinolones resulted in the development of various fluoroquinolone agents, namely norfloxacin, ofloxacin, ciprofloxacin, levofloxacin, and moxifloxacin [17,18]. Norfloxacin, ofloxacin, and ciprofloxacin have antibacterial effects against gram-negative bacteria, while levofloxacin (a stereoisomer of ofloxacin) is active against gram-negative and gram-positive bacteria. Moxifloxacin is mainly active against gram-positive pathogenic bacteria [18].

Fluoroquinolones have been applied to treat various bacterial infections, including urinary tract, respiratory tract, and enteric infections. However, human pathogen bacteria can develop resistance to fluoroquinolones through various mechanisms. The main mechanism of fluoroquinolone resistance is the accumulation of mutations in gyrase- and topoisomerase IV-coding genes (referred as quinolone-resistance-determining regions/QRDR) [19,20]. Additionally, in gram-negative pathogens, plasmid-mediated quinolone resistance (PMQR) determinants enhance development of fluoroquinolone resistance. PMQRs are represented by Qnr determinants, QepA and OqxAB efflux pumps, and aminoglycoside-acetyltransferase Ib-c variants [21,22,23]. 

Based on widespread fluoroquinolone resistance, the clinical use of earlier fluoroquinolones has been limited [24,25]. However, new developments in fluoroquinolones have resulted in novel chemical structures and improved antibacterial efficacy.

## 3. Delafloxacin

**Figure 1:**
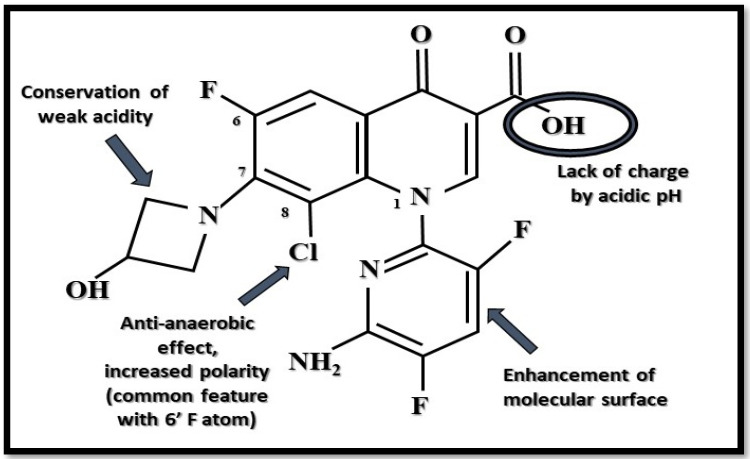
Unique anionic structure of delafloxacin by acidic pH. Arrows show the function of the given substituents. In the position N1, a heteroaromatic substituent provides a larger molecular surface than that of other fluoroquinolones (Figure was created by Dániel Gulyás).

Delafloxacin (former terms were ABT-492, *RX-3341*, and *WQ-3034*) is currently the only available anionic (non-zwitterionic) fluoroquinolone which targets both bacterial DNA gyrase and topoisomerase IV enzymes of gram-positive and gram-negative bacteria with equal affinity [26,27,28]. Its chemical structure is 1-(6-amino-3,5-difluoro-2-pyridinyl)-8-chloro-6-fluoro-7-(3-hydroxy-1-azetidinyl)-4-oxo-1,4-dihydro-3-quinolinecarboxylate [18] (Figure 1). It has a special chemical structure which makes it a weak acid. It stays uncharged by acidic pH, which aids its transmembrane transfer into the bacterial cell, where it accumulates. In intracellular space (neutral pH), it maintains its anionic deprotonated appearance and concentration-dependent antimicrobial activity [16,29]. Based on this unique anionic property, it has an increased antibacterial effect in acidic (inflammatory) environments, for example, in phagolysosomes; in biofilm, as well as in skin; in soft tissue; and in abscesses. Additionally, it has a heteroaromatic substitution which provides a larger molecular surface, and a chlorine atom, which enhances its efficacy against anaerobic bacteria and provides strong polarity. This larger molecular surface can enhance antibacterial activity against strains that are resistant to commonly used fluoroquinolones [24,29,30] (Figure 1).

Delafloxacin is a bactericidal agent, and it can be given per os or by intravenous infusion; switch dosing is also accepted. By oral therapy, a 450 mg dose is needed in order to reach a corresponding concentration-time profile, compared to an intravenous 300 mg dose. In rare cases, oral bioavailability (58.8%) can be decreased through pharmacological interactions with other medications that contain multivalent metal cations, such as Al^3+^, Mg^2+^, Fe^2+^, or Zn^2+^, because of chelation effects [29,31]. In addition, delafloxacin does not inhibit cytochrome P450 isoenzymes; consequently, it does not cause clinically relevant drug–drug interactions with most commonly used medications. Delafloxacin did not show potential synergistic or antagonistic effects with other antibiotics [16,24,32]. Plasma protein binding of delafloxacin is approximately 84% [24]. Patients with kidney failure received a higher dose of this antibiotic, but its activity was not significantly affected by hepatic impairment [32,33].

All known fluoroquinolones may provoke several side effects, such as tendinitis, tendon rupture, photosensitivity, neurological symptoms, and exacerbations of myasthenia gravis, with muscle weakness and QT interval prolongation. In the case of delafloxacin, the FDA has reported peripheral neuropathy, hypersensitivity, and *Clostridium difficile*-associated diarrhea as possible, but less severe compared to other fluoroquinolones, dose-dependent adverse effects. Of these symptoms, the most frequent treatment-emergent adverse effects (TEAEs) were diarrhea, vomiting, and infusion site extravasation. As uncommon complications, hyperglycemic episodes (one patient of ten was affected) and serious elevation of transaminase enzymes (in one patient of twenty-two) were mentioned in these studies. On the other hand, they also demonstrated a lack of photosensitivity and cardiotoxicity, so all in all it appears to be a well-tolerated antibiotic [16,24,30,34,35,36,37]. Delafloxacin is not recommended during pregnancy, although side effects during lactation and teratogenic effects of delafloxacin were not found in animal studies. It is also contraindicated for pediatric use [16,30].

Delafloxacin has a broad antibacterial spectrum, both in vitro and in vivo, against a wide range of bacteria: gram-positive cocci (staphylococci, including methicillin-resistant *S. aureus* (MRSA) and methicillin-sensitive *S. aureus* (MSSA) isolates, streptococci, and enterococci), gram-negatives (e.g., *P.aeruginosa, Acinetobacter* sp., *Haemophilus influenzae*, *Moraxella catarrhalis*, extended-spectrum beta-lactamase (ESBL) producing *Escherichia coli* and *K. pneumoniae*, *Neisseria gonorrhoeae*, and *Helicobacter pylori*), anaerobes (e.g., *Bacteroides fragilis*), and causative agents of atypical pneumonia, including *Legionella pneumophila*, *Chlamydia pneumoniae*, and *Mycoplasma pneumoniae* [26,27,38,39,40,41,42]. Based on this broad spectrum of antibacterial activity, current guidelines approved delafloxacin for treatment of adults’ acute bacterial skin and skin-structure infections (ABSSSI), as well as community-acquired bacterial pneumonia (CABP) [43,44,45] (Table 1, Table 2 and Table 3). According to its antipseudomonal effect, delafloxacin is also a promising agent to treat fluoroquinolone-resistant *P.aeruginosa* lung infections in patients with cystic fibrosis. Furthermore, the enhanced antibacterial efficacy of delafloxacin in acidic environment enables it for therapeutic use against *H. pylori* infections [42,46]. 

Compared to the other group members, delafloxacin has increased stability against bacterial gene mutations of DNA gyrase (*gyrA* and *gyrB*) and topoisomerase IV (*grlA* and *grlB* in gram-positive bacteria; *parC* and *parE* in gram-negatives) [30]. Based on the double-targeting feature of these enzymes, double- or triple-point mutations of QRDR are required for development of resistance. Interestingly, resistance selection studies were performed, and delafloxacin-resistant strains were selected out from previously susceptible MRSA isolates, by development of mutations in *gyrA* or *gyrB* [26,47]. Moreover, delafloxacin-resistant MRSA clinical isolates were obtained from healthcare-associated infections in seven hospitals in Brooklyn (New York) [48]. Delafloxacin-resistant gram-negative pathogens were also reported, and strains exhibiting high-level ciprofloxacin resistance tended to also show delafloxacin resistance. Ciprofloxacin and delafloxacin-resistant *E. coli*, *Enterobacter* spp., *P. aeruginosa*, and *A. baumannii* were identified [28]. In the case of *N. gonorrhoeae*, a selection of delafloxacin-resistant mutants was performed. In the delafloxacin-resistant strains, QRDR mutations were associated with upregulation of MtrC-, MtrD, MtrE-, and NorM-efflux pumps [39].

**Table 1:** Comparison of the clinically relevant features of novel fluoroquinolones.

Novel Fluoroquinolones	Delafloxacin	Finafloxacin	Zabofloxacin	Reference
**Chemical structure**	Unique anionic (non-zwitterionic) structure, with special substituents and augmented polarity.	Zwitterionic chemical structure of fluoroquinolones supplemented with substituents.	Zwitterionic chemical structure of fluoroquinolones supplemented with substituents (two forms are available).	[16,18,29]
**Bioavailability**	58.8%	75% (by oral use)	No data available.	[29,31]
**Protein binding**	Approximately 84%	No data available.	No data available.	[24]
**Mechanism of action**	Dual-targeting of DNA gyrase and topoisomerase IV enzymes of gram-positives and gram-negatives with equal affinity. Increased bactericidal effect in acidic pH	Dual-targeting (weaker effect compared to other group members) of DNA gyrase and topoisomerase IV enzymes of gram-positives and gram-negatives with equal affinity. Increased bactericidal effect in acidic pH.	Dual-targeting of DNA gyrase and topoisomerase IV enzymes, predominantly of community-acquired respiratory tract pathogen gram-positives, and some gram-negatives. Ineffective against major nosocomial gram-negatives.	[26,27,28,49,50]
**Approved Indication**	Acute bacterial skin and skin-structure infections (ABSSSI) of adults caused by MRSA, MSSA, *S.haemolyticus*, *S. lugdunensis*, *S. agalactiae*, *Streptococcus anginosus Group*, *S. pyogenes*, *E. faecalis*, *E. coli*, *E. cloacae*, *K.pneumoniae*, and *P.aeruginosa*. Community-Acquired Bacterial Pneumonia of adults caused by *S.pneumoniae*, MSSA, *K. pneumoniae*, *P.aeruginosa*, *H. influenzae*, *H. parainfluenzae*, *C. pneumoniae*, *L. pneumophila*, and *M. pneumoniae*.	Otic suspension for acute otitis externa caused by *P. aeruginosa* and *S. aureus* in patients age one year and older.	Oral administration for acute bacterial exacerbation of chronic obstructive pulmonary disease (COPD).	[18,43,44,45,51]

**Table 2:** Other features of novel fluoroquinolones.

Novel Fluoroquinolones	Delafloxacin	Finafloxacin	Zabofloxacin	Reference
**Further possible clinical applications**	*P. aeruginosa*-mediated lung infections in patients with cystic fibrosis. Infection by multidrug-resistant *H.pylori*.	Complicated and non-complicated urinary-tract infections. Zoonoses, e.g., *Y.pestis* and *B.anthracis*. Prophylaxis and treatment of *B. pseudomallei* infections.	Community-acquired bacterial pneumonia.	[18,42,46,50]
**Contraindication and** **side effects**	Well-tolerated; lack of teratogenic effect, photosensitivity and cardiotoxicity. Diarrhoea, vomiting and other fluoroquinolone-specific adverse affects may occur.	Ophthalmic use is contraindicated. In animal studies, showed teratogenic ability and fluoroquinolone-specific adverse effects (per os). Hypersensitivity and pruritus.	Well-tolerated; lack of long QT-syndrome; in animal studies, subacute toxicity (atrophy of endocrine organs with vomitus by dogs) was found. Mainly gastrointestinal adverse effects were reported.	[16,18,24,30,34,35,36,37,51]
**Resistance mechanisms**	Multiple mutations by bacterial topoisomerase IV enzymes. Single mutations with efflux pumps. Generally fluoroquinolone-resistant strains are susceptible to to Delafloxacin (cross-resistance is also known).	Multiple mutations in bacterial topoisomerase IV enzymes. Cross-resistance with other fluoroquinolones was reported.	Multiple mutations in bacterial Topoisomerase IV enzymes. Generally fluoroquinolone-resistant strains are susceptible to Zabofloxacin.	[18,26,28,30,39,47,48,51]

**Table 3:** MIC values of novel fluoroquinolones in the case of ESKAPE group members and other pathogens. For finafloxacin the table contains values in slightly acidic pH (5.8–6.2), and for zabofloxacin it demonstrates values of fluoroquinolone-resistant MRSA strains.

Novel Fluoroquinolones	Delafloxacin		Finafloxacin		Zabofloxacin		Reference
	MIC_90_ (mg/L)	MIC Range	MIC_90_ (mg/L)	MIC Range	MIC_90_ (mg/L)	MIC Range	
*E. faecalis*	1	≤0.004 to 2	16	0.25–16	2	0.008 ≥ 4	[18,24,38,52]
*E. faecium*	>4	0.008 to > 4	No data available.	0.5–32	16	2–32	[18,24,38,52]
**MRSA**	0.5	≤ 0.004 to 4	0.125	0.06–0.125	32	0.016–0.64	[18,24,38,52]
**MSSA**	0.008		No data available.		No data available.		[24,38]
*E. coli*	>4	0.008 to > 4	32	2–64	1	0.015–64	[18,24,38,52]
*K.pneumoniae*		0.06 to > 4	0.5	0.008–1		0.06–8	[24,38,52]
*P. aeruginosa*		0.015 to > 4	2	0.25–8	8	0.125–32	[24,38,52]
*A. baumannii/* *A. calcoaceticus*			No data available.		4	0.008–8	[30]
*S. maltophilia*	2	0.12–16	1	0.125–16	No data available.		[30,52]

## 4. Finafloxacin

**Figure 2:**
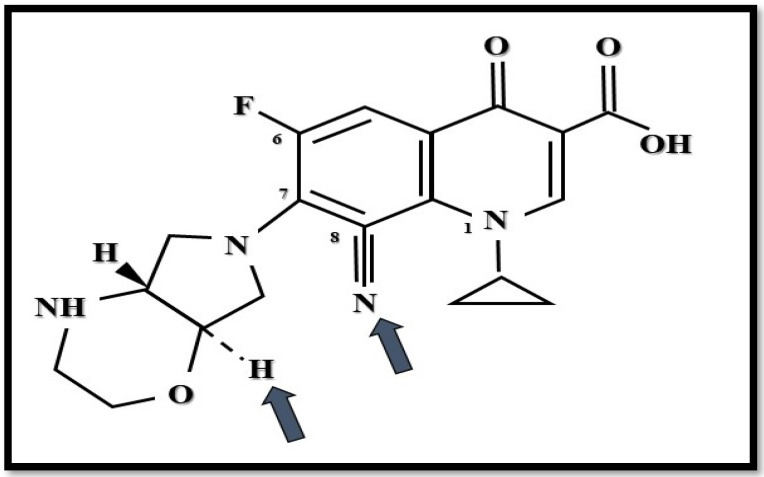
Structure of finafloxacin. Arrows show novel substituents compared to other fluoroquinolones (Figure was created by Dániel Gulyás).

Finafloxacin (former term: BAY35-3377) has a zwitterionic chemical structure with a chiral cyano-substituent and pyrrolo-oxazinyl component [18] (Figure 2). Based on this chemical construction, it is characterized by the dual inhibition of both DNA gyrase and topoisomerase IV enzymes, which leads to broad-spectrum antibacterial activity [49,50].

Finafloxacin shows increased antibacterial activity in acidic environments (e.g., in an infection site), although in neutral pH it possesses a similar bactericidal effect compared to other clinically used fluoroquinolones [49].

Although the FDA primarily indicated finafloxacin as a 0.3% otic suspension for therapy of acute otitis externa, caused by *P. aeruginosa* and *S. aureus*, in patients aged one year and older, [51] finafloxacin has remarkable antibacterial efficacy against major gram-negative bacteria, including fluoroquinolone-resistant *Enterobacteriaceae*, *A.baumannii*, and *L. pneumophila.* Major gram-positive pathogens show finafloxacin susceptibility, for example, MRSA and un-phagocytized *Listeria monocytogenes* [52,53,54].

Evaluation of the pharmacokinetic parameters of finafloxacin showed that, after oral doses (400 to 800 mg), it reached higher concentrations in urine than the measured MIC values of the most important causative agents of complicated UTIs, including *E.coli*, *P.aeruginosa*, and even fluoroquinolone-resistant uropathogens. In this case, bioavailability reached 75% [55,56,57]. Based on these results, many studies suggest finafloxacin as a suitable antibiotic for therapy of difficult-to-treat UTIs in the future [50,55,56,57,58]. Furthermore, because of its pH-related activity, it has a strong effect against intracellular pathogens (e.g., *Coxiella burnetii* [59], *Burkholderia pseudomallei*, *Yersinia pestis*, *Francisella tularensis*, and *Bacillus anthracis*). These are causative agents of severe zoonotic diseases [60,61,62]. According to different studies, finafloxacin was also able to overcome the efflux-pumps of fluoroquinolone-resistant *B. pseudomallei* strains, so it is a potential antibiotic for melioidosis prophylaxis and therapy [63,64,65] (Table 2).

Due to these favorable features, despite the well-known adverse effects of oral fluoroquinolones, finafloxacin appeared as a teratogenic antimicrobial agent in animal studies. As an eardrop, allergic reactions and pruritus may also occur. Based on this local form of clinical use, only a minimal concentration was detected in blood; consequently, relevant drug–drug interactions were not found. Cross-resistance between finafloxacin and the earlier generations of fluoroquinolones was also observed. Mainly, the chromosomal mutations of QRDR and drug efflux mechanisms play an important role in development of resistance against this novel antibiotic [51].

## 5. Zabofloxacin

**Figure 3:**
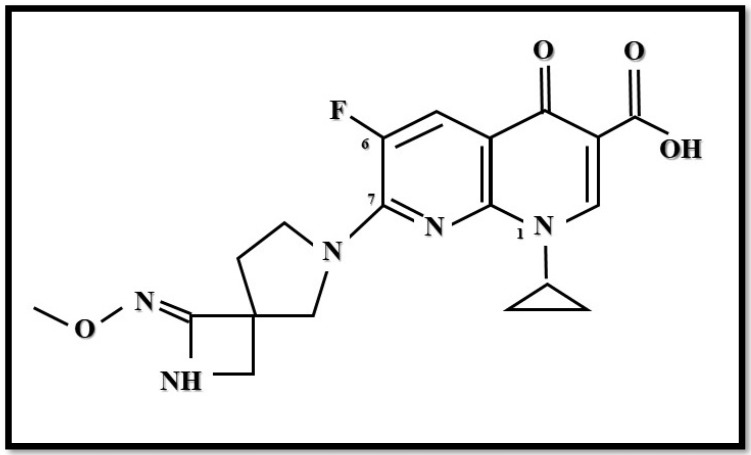
Structure of zabofloxacin (Figure was created by Dániel Gulyás).

Zabofloxacin (DW-224a) is an orally administered broad-spectrum fluoroquinolone (fluoronaphthyridone) that targets both DNA gyrase and topoisomerase IV enzymes. Its chemical structure is {1-cyclopropyl-6-fluoro-7-[8-(methoxyimino)-2,6-diazaspiro[3,4]oct-6-yl]-4oxo-1,4-dihydro[1,8]naphthyridine-3-carboxylic acid hydrochloride} (Figure 3). There are two forms of zabofloxacin available: zabofloxacin hydrochloride (DW-224a) and aspartate (DW-224aa) [66,67].

Zabofloxacin was approved for clinical use in South Korea, the Middle-East, and North-African countries [68,69]. The indication of zabofloxacin is in treatment of patients with acute bacterial exacerbation of chronic obstructive pulmonary disease.

Zabofloxacin demonstrates bactericidal effects against major respiratory tract pathogens, namely *Streptococcus pneumoniae*, *Staphylococcus aureus*, *Haemophilus influenzae*, and *Moraxella catarrhalis*. Furthermore, zabofloxacin showed bactericidal effects against *Neisseria gonorhoeae*, as well as against fluoroquinolone-resistant *S. aureus*. Additionally, zabofloxacin was equally effective against *Klebsiella pneumoniae* compared to moxifloxacin. However, zabofloxacin lacks potency against major nosocomial gram-negative pathogens, namely *Pseduomonas aeruginosa* and *Acinetobacter baumannii* [66,70,71,72,73] (Table 3).

Zabofloxacin was found to be a well-tolerated agent in healthy male volunteers. Among the detected adverse events were nausea (7% of individuals), hypotension (3%), somnolence (3%), and increase of blood phosphokinase (3%) [74].

Several clinical studies were conducted to test the antibacterial efficacy and pharmacokinetic profile of zabofloxacin. A phase one clinical trial investigated the pharmacokinetic profile of 183 mg and 367 mg zabofloxacin, compared to 250 mg levofloxacin (ClinicalTrials.gov identifier: NCT02212795). The pharmacokinetic characteristics of zabofloxacin hydrochloride (DW224a) and zabofloxacin aspartate (DW-224aa) were investigated in a phase one clinical trial (ClinicalTrials.gov identifier: NCT01341249). A phase three clinical trial was conducted to compare 367 mg zabofloxacin with 400 mg moxifloxacin in therapeutic use (ClinicalTrials.gov identifier: NCT01658020). A phase two clinical trial was also organized to test the safety and efficacy of zabofloxacin in community acquired pneumonia, but this trail was terminated. No results were posted (ClinicalTrials.gov identifier: NCT01081964). Zabofloxacin hydrochloride and zabofloxacin aspartate were analyzed in the phase one clinical trial. A total of twenty-nine healthy male individuals were enrolled in a random, open-label single-dose study, and pharmacokinetic characteristics were investigated. During this trial, orally-administered 366.7 mg zabofloxacin hydrochloride and 366.5 mg zabofloxacin aspartate were analyzed. The peak serum concentration (C_max_) values were 1.9 ± 0.5 mg/L and 2 ± 0.3 mg/L, and these values were reached between 0.5–4 h and 0.8–3 h, respectively. The half-life time of zabofloxacin was 8 ± 1 h for both formulations [74]. The toxicity of zabofloxacin hydrochloride (366.7 mg) and zabofloxacin aspartate (366.5 mg) was also investigated. Both formulations were well tolerated in healthy male individuals in a phase one clinical trial. Only some adverse events were recorded; notably, nausea, hypotension, somnolence, and increased blood phosphokinase. However, prolongation of QT intervals was not detected [74]. The efficacy of oral zabofloxacin (367 mg once daily for 5 days) was compared with moxifloxacin (400 mg once daily for 7 days) in a phase three, multicenter, double-blinded, randomized non-inferiority clinical trial [75]. In this study, a total of 345 patients diagnosed with chronic obstructive pulmonary disease, with moderate exacerbations, were included. The overall clinical cure rates were 88.2%, for zabofloxacin, and 89.1% for moxifloxacin. There was no statistically significant difference (*p* = 0.89) detected. According to this clinical trial, zabofloxacin reaches equal clinical outcome, compared to that of moxifloxacin, by the end point of the test period. The antibacterial efficacy of zabofloxacin was compared with that of moxifloxacin, in a patient group suffering from lower-respiratory-tract infections, without chronic bronchitis. In this study, 85.9% and 84.2% cure rates were observed, respectively, and no statistically significant differences were reported (*p* = 0.76) [75].

## 6. Conclusions

Nowadays, antibiotic-resistant bacteria can cause severe infections, including nosocomial and community-acquired diseases. The ease with which human pathogen bacteria develop resistance against commonly used antibiotics is worrisome. Rapid dissemination of antibiotic-resistant bacteria is detected all over the world, and infections caused by these bacteria are associated with high mortality rates. Thus, therapy of these infections is a great challenge worldwide. The number of available effective antimicrobial agents is limited. In the last few years, some novel antimicrobial agents were introduced into clinical practice, but further developments are still required [10].

Three new fluoroquinolones, delafloxacin, finafloxacin, and zabofloxacin, have special chemical structures which lead to many favorable properties; for example, broad antibacterial spectrums, good tissue penetration, and the lack of severe adverse effects. On the other hand, development of resistance against these new fluoroquinolones can occur; therefore, the prudent use of antibiotics is necessary. These new agents are only approved to treat certain infections, such as adults’ acute bacterial skin and skin-structure infections, community-acquired pneumonia, and *P. aeruginosa* related acute otitis externa or acute bacterial exacerbation of chronic obstructive pulmonary disease. Since their first clinical use, new studies have recommended other possible indications for these antibiotics, which can further enlarge their clinical applications. These further possible indications include the treatment of urinary-tract infections and lower-respiratory-tract infections. However, in the meantime, some delafloxacin-resistant *S. aureus* clinical isolates were also reported [48], as well as reports of delafloxacin-resistant *E.coli*, *Enterobacter* spp., *P. aeruginosa*, and *A. baumannii* [28]. These are alarming cases, and indicate a stronger need for the prudent use of antibiotics.

In summary, the introduction of novel antimicrobial agents provides new options to treat infections caused by bacteria that are already resistant to commonly used antibiotics. Continuous monitoring of the development of resistance against these novel antibiotics is necessary, and the prudent use of antibiotics is also important for successful treatment in the future.
